# Design, Synthesis and Biological Evaluation of Novel *N*-Pyridyl-Hydrazone Derivatives as Potential Monoamine Oxidase (MAO) Inhibitors

**DOI:** 10.3390/molecules23010113

**Published:** 2018-01-08

**Authors:** Gülhan Turan-Zitouni, Weiam Hussein, Begüm Nurpelin Sağlık, Aouatef Tabbi, Büşra Korkut

**Affiliations:** 1 Department of Pharmaceutical Chemistry, Faculty of Pharmacy, Anadolu University, 26470 Eskişehir, Turkey; white.wf.rose@gmail.com (W.H.); bnsaglik@anadolu.edu.tr (B.N.S.); awateftabbi@yahoo.fr (A.T.); 2 Department of Pharmaceutical Chemistry, Faculty of Pharmacy, Aden University, 6075 Aden, Yemen; 3 Doping and Narcotic Compounds Analysis Laboratory, Faculty of Pharmacy, Anadolu University, 26470 Eskişehir, Turkey; 4 Department of Chemistry, Faculty of Sciences, Mentouri University, 325 Constantine, Algeria; 5 Department of Pharmaceutical Toxicology, Faculty of Pharmacy, Anadolu University, 26470 Eskişehir, Turkey; busrakorkut@anadolu.edu.tr

**Keywords:** MAO inhibitors, *N*-pyridyl-hydrazone derivatives, inhibitory potency, enzyme kinetic studies

## Abstract

A new series of *N*-pyridyl-hydrazone derivatives was synthesized by using a simple and efficient method. The final compounds obtained were screened for their inhibitory potency against monoamine oxidase (MAO) A and B. The newly synthesized compounds **2a**–**2n** specifically inhibited monoamine oxidases, displaying notably low IC_50_ values. Compounds **2i** and **2j**, with a CF_3_ and OH group on the 4-position of the phenyl ring, respectively, showed considerable MAO-A and MAO-B inhibitory activities. Compounds **2k**, **2l** and **2n**, with *N*-methylpyrrole, furan and pyridine moieties instead of the phenyl ring, were the most powerful and specific inhibitors of MAO-A, with IC_50_ values of 6.12 μM, 10.64 μM and 9.52 μM, respectively. Moreover, these active compounds were found to be non-cytotoxic to NIH/3T3 cells. This study supports future studies aimed at designing MAO inhibitors to obtain more viable medications for neurodegenerative disorders, such as Parkinson’s disease.

## 1. Introduction

MAO exists in two isoforms, MAO-A and MAO-B, which have contrasting substrate specificity; MAO-A specifically deaminates serotonin and norepinephrine, and MAO-B acts specifically on phenylethylamine. Given the essential role of MAOs in the inactivation of neurotransmitters, the mechanism of action of MAO-A and MAO-B have been an imperative focus for the treatment of the pathologies of many neurodegenerative diseases. In particular, MAO-B inhibitors are used as a part of the treatment for Alzheimer’s and Parkinson’s diseases, whereas MAO-A inhibitors are used as antidepressants and antianxiety agents [1,2]. Therefore, investigations on MAO inhibitors have increased in the recent years owing to their restorative effect on mental health. For example, specific MAO-A inhibitors such as iproniazid, clorgyline and moclobemide exert invigoration and antianxiety action [3,4]. Specific MAO-B inhibitors, for example selegiline, rasagiline and lazabemide, complement treatments for Parkinson’s and Alzheimer’s disease [5]. Unfortunately, the vast majority of the currently used MAO inhibitors, such as iproniazid and tranylcypromine, appear to initiate hepatotoxicity and other critical symptoms, such as the “cheese reaction” [6,7]. Therefore, there is an urgent need for the development of novel MAO inhibitors for neuro-related diseases. It is well known that, the substrates and inhibitors of MAOs usually carry an amino or imino group, which appears to play an essential role in the orientation and complex formation at the active site of the enzyme. Numerous substituted hydrazines and hydrazides have been studied as MAO inhibitors [8,9]. Furthermore, some studies have confirmed particular moieties, including a pyridine nucleus, to possess an inhibitory effect on MAO [10,11]. Based on these investigations, in this study, we aimed to design and synthesize a novel series of *N*-pyridyl-hydrazone derivatives **2a**–**2n** and report their inhibitory effects on MAO-A and MAO-B activities.

## 2. Results and Discussion

### 2.1. Chemistry

The 2-[(2-substitutedbenzylidene)hydrazinyl]-6-ethoxy-3-nitropyridine derivatives **2a**–**2n** were synthesized by using a simple previously reported method [12,13] and in accordance with a protocol used in our earlier research. The structures of the synthesized compounds were completely identified by spectral data, as reported in the experimental section. In the IR spectra, strong bands at 1600 and 1400 cm^−1^ appeared, due to the presence of the C=N and C=C groups, respectively. In the ^1^H-NMR spectra, we clearly observed the presence of C=N protons at 8.00–8.90 ppm. The aromatic protons appeared at their expected values. In the ^13^C-NMR spectra, these compounds displayed the predicted peak values, but it should be noted that owing to poor solubility of compounds **2m** and **2n**, we were unable to record their ^13^C-NMR spectra. Molecular ion peaks, in conjunction with the base peaks in the mass spectra, provided additional confirmation for the verification of the structures of the newly synthesized compounds.

### 2.2. Inhibition Potency of the Compounds

**Figure 1:**
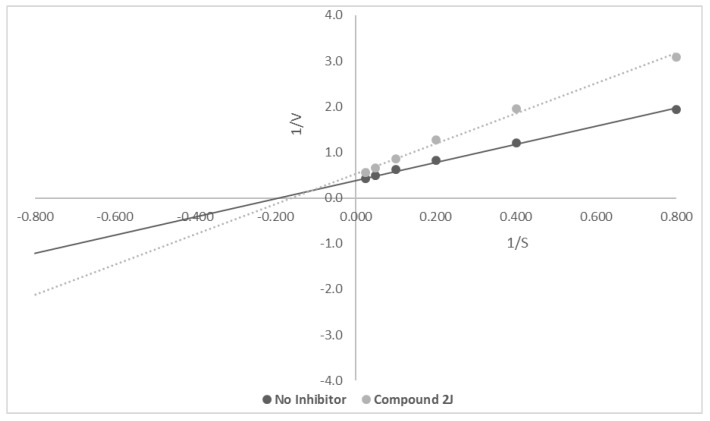
Lineweaver–Burk plots on MAO-A enzyme for compound **2j** (IC_50_ = 6.25 μM). Substrate (kynuramine) concentrations used: 40, 20, 10, 5, 2.5 and 1.25 μM. 1/V: 1/rate of reaction [1/(nmoles/min/mg protein)], 1/S: 1/substrate concentration (1/µM).

Our results indicated that a large number of the synthesized compounds have powerful inhibitory effects against both MAO-A and MAO-B, with IC_50_ concentrations in the micromolar range; thus, the effects following introduction of substituents of various sizes to the phenyl ring were evaluated. For compounds **2i** and **2j**, the incorporation of CF_3_ and OH groups on the 4-position of the phenyl ring, respectively, showed massive MAO-A and MAO-B inhibitory activities. It is well known that most of the monoamine oxidase inhibitors (MAO) belong to a group of nitrogen heterocycles, such as pyrazoline, indole, xanthine, oxadiazole, benzimidazole, pyrrole, quinoxaline, thiazole, with other related heterocycles also being reported [14]. As expected, our newly synthesized compounds with heterocyclic moieties showed strong inhibitory activity. Thus, compounds **2k**, **2l** and **2n** with *N*-methylpyrrole, furan and pyridine moieties, instead of the phenyl ring, were the most powerful and specific inhibitors against MAO-A, with IC_50_ values 6.12 μM, 10.64 μM and 9.52 μM, respectively. The screening results indicated that some of the tested compounds inhibited MAO-B. Compound **2j** was found to be more potent and specific activity toward MAO-B, with an IC_50_ of 9.30 μM. Therefore, we determined that compound **2j** was a successful inhibitor of both MAO-A and MAO-B. Considering the closeness to the standard, compound **2l** demonstrated an extraordinary level of MAO-A inhibitory activity. The MAO-A enzyme kinetics of the most active compound, **2j**, were also studied. The nature of MAO-A inhibition caused by this compound was investigated by using the graphical analysis of steady-state inhibition data (Figure 1). The reciprocal plots (Lineweaver–Burk plots) identified compound **2j** as a mixed type inhibitor owing to the different intercepts on both the *y*- and *x*-axes. The values of K_m_ and V_max_ were calculated by nonlinear regression based on a previous study [15], and found to be 0.157 μM and 1.903 nmol/min/mg protein, respectively. According to our results, these newly synthesized compounds may be considered as promising therapeutic agents for neurodegenerative and stress-related disorders; however, further trials on the selectivity and effects on other related enzymes should be performed.

### 2.3. Cytotoxicity Test

Toxicity is the main reason for drug candidate failure at all stages of the new drug development process. The major part of safety-related attrition occurs at preclinical phases while predicting preclinical safety liabilities earlier in the drug development process. This strategy enables the design and/or selection of improved drug candidates that have more possibilities to become commercialized drugs. Therefore, the MTT test was conducted in NIH/3T3 mouse embryonic fibroblast cell lines (ATCC CRL1658), which is the ISO-prescribed cell line for cytotoxicity screening (10993-5, 2009) [16]. The IC_50_ values of compounds **2i**, **2j**, **2k**, **2l** and **2n** (for all; >1000 µM) against NIH/3T3 cells were higher than their IC_50_ values against MAO. Thus, the cytotoxicity test results confirmed the importance of compounds **2i**, **2j**, **2k**, **2l** and **2n** as MAO inhibitors.

### 2.4. Molecular Docking Studies

**Figure 2:**
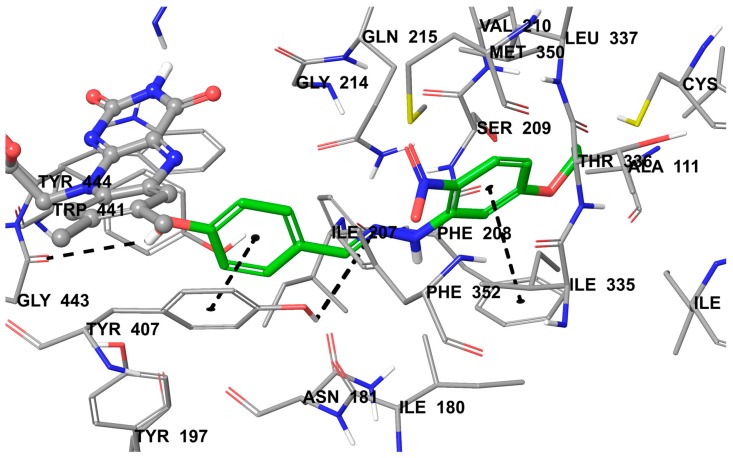
The interacting mode of compound **2j** in the active region of *h*MAO-A. The inhibitor and the important residues in the active site of the enzyme are presented by tube model. The FAD molecule is colored grey with ball and stick model.

To determine the binding modes of compound **2j**, docking studies were performed by using the Maestro interface. The X-ray crystal structure of *h*MAO-A, obtained from the Protein Data Bank server (www.pdb.org), was used (PDB ID: 2Z5X) [17]. The docking pose of compound **2j** is presented in Figure 2. 

It can be observed that compound **2j** is located in close proximity to the FAD cofactor and binds tightly to the amino acid residues in the cavity. As known by the docking pose, compound **2j** interacts with the active site by establishing two hydrogen bonds and two π-π interactions. The first π-π interaction was observed between three substituted benzene rings and the phenyl of Phe208. The other phenyl ring in the structure interacted with the phenyl of Tyr407 by π-π interaction. The imine nitrogen of the hydrazone moiety established a hydrogen bond with the hydroxyl of Tyr407, whereas the other hydrogen bond was established between the hydroxyl *para*-substituted phenyl and the carbonyl of Gly443. Collectively, these interactions provide an explanation the inhibitory activity of compound **2j**. 

## 3. Materials and Methods

### 3.1. Chemistry

**Scheme 1:**
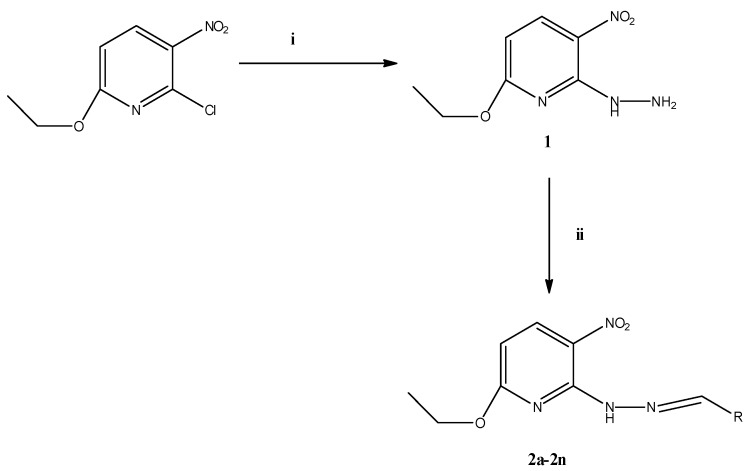

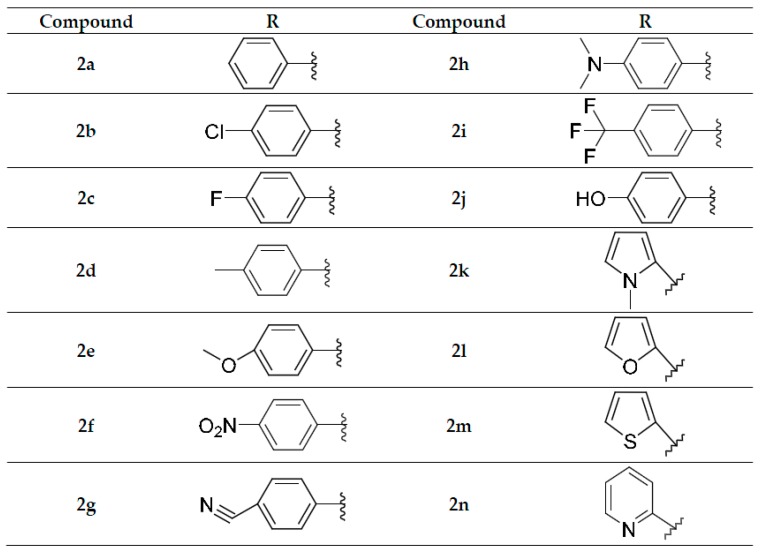
The synthesis of compounds **2a**–**2n**. *Reagents and Conditions*: (i) 2-chloro-6-ethoxy-3-nitropyridine (0.02 mol/2.5 g) and hydrazine, ethanol stirred at room temperature for 3 h; (ii) equimolar quantities of hydrazides and different substituted benzaldehydes (0.003 mol), ethanol, refluxed for 5 h.

All compounds and chemicals were purchased from Sigma-Aldrich Chemicals (Sigma-Aldrich Corp., St. Louis, MO, USA) or Merck KGaA (Darmstadt, Germany). The melting points of the synthesized compounds were assessed by using a MP90 digital melting point apparatus (Mettler Toledo, Ohio, OH, USA) and were uncorrected. The ^1^H-NMR spectra and ^13^C-NMR spectra were recorded by using a Bruker 300 MHz digital FT-NMR spectrometer (Bruker Bioscience, Billerica, MA, USA) and collected in DMSO-*d*_6_ (Supplementary Materials). The IR spectra were obtained by using a Shimadzu IR Prestige-21 instrument (Shimadzu, Kyoto, Japan). The LC-MS/MS studies were conducted by using a Shimadzu 8040 LC-MS/MS system (Shimadzu, Kyoto, Japan). The purities of the compounds were checked by TLC on silica gel 60 F254 (Merck KGaA, Darmstadt, Germany). The general synthesis method is presented in Scheme 1.

### 3.2. Synthesis of the Compounds

#### 3.2.1. Preparation of 6-Ethoxy-2-hydrazinyl-3-nitropyridine (**1**)

A mixture of 2-chloro-6-ethoxy-3-nitropyridine (0.02 mol/2.5 g) and hydrazine (2 mL) in ethanol was stirred at room temperature for 3 h and filtered. The residue was recrystallized from ethanol [18,19].

#### 3.2.2. Preparation of 2-[(2-Substitutedbenzylidene)hydrazinyl]-6-ethoxy-3-nitropyridines **2a**–**2n**

Equimolar quantities of hydrazides (0.003 mol/0.75 g) and 0.003 mol of different substituted benzaldehydes in ethanol (15 mL) were refluxed for 5 h. The reaction mixture was then cooled and the precipitated solid was filtered and recrystallized from ethanol.

*2-(2-Benzylidenehydrazinyl)-6-ethoxy-3-nitropyridine* (**2a**). Yield: 84%, Melting point: 168 °C, ^1^H-NMR (300 MHz, DMSO-*d*_6_) δ (ppm): 1.49 (3H, t, CH_3_), 4.55 (2H, q, CH_2_), 6.37 (1H, d *J* = 9.02 Hz, pyridine-H), 7.42–7.51 (3H, m, Ar-H), 7.60 (2H, d *J* = 6.88 Hz, Ar-H), 8.40 (1H, d *J* = 9.03 Hz, pyridine-H), 8.60 (1H, s, N=CH), 11.68 (1H, s, NH). ^13^C-NMR (75 MHz, DMSO-*d*_6_, ppm): 14.77 (CH_3_), 63.49 (CH_2_), 103.19 (Ar-CH), 121.99 (Ar-C), 127.50 (2CH, Ar-), 129.38 (2CH, Ar-), 130.49 (Ar-CH), 134.99 (Ar-C), 138.62 (Ar-CH), 148.44 (N=CH), 149.57 (C-NH), 166.5 (C-OC_2_H_5_). IR (cm^−1^): ν_max_ 3370, 3315 (N-H stretching), 1600–1430 (C=N and C=C stretching), 1230–1012 (C-O). MS [M + 1]^+^: *m*/*z* 287.07.

*2-[2-(4-Chlorobenzylidene)hydrazinyl]-6-ethoxy-3-nitropyridine* (**2b**). Yield: 88%, Melting point: 211 °C, ^1^H-NMR (300 MHz, DMSO-*d*_6_) δ (ppm): 1.43 (3H, t, CH_3_), 4.52 (2H, q, CH_2_), 6.38 (1H, d *J* = 9.02 Hz, pyridine-H), 8.00 (2H, d *J* = 8.80 Hz, Ar-H), 8.33 (2H, d *J* = 8.79 Hz, Ar-H), 8.40 (1H, d *J* = 8.98 Hz, pyridine-H), 8.70 (1H, s, N=CH), 11.85 (1H, s, NH). ^13^C-NMR (75 MHz, DMSO-*d*_6_, ppm): 14.85 (CH_3_), 63.60 (CH_2_), 103.21 (Ar-CH), 121.90 (Ar-C), 124.95 (2CH, Ar-), 128.18 (2CH, Ar-), 130.49 (Ar-CH), 134.19 (Ar-C), 149.00 (N=CH), 149.55, (C-NH), 166.34 (C-OC_2_H_5_). IR (cm^−1^): ν_max_ 3355, 3312 (N-H stretching), 1655–1420 (C=N and C=C stretching), 1222–1000 (C-O). MS [M + 1]^+^: *m*/*z* 322.11.

*6-Ethoxy-2-[2(4-fluorobenzylidene)hydrazinyl]-3-nitropyridine* (**2c**). Yield: 82%, Melting point: 191 °C, ^1^H-NMR (300 MHz, DMSO-*d*_6_) δ (ppm): 1.38 (3H, t, CH_3_), 4.53 (2H, q, CH_2_), 6.38 (1H, d J = 9.07 Hz, pyridine-H), 7.55 (2H, d *J* = 8.64 Hz, Ar-H), 7.77 (2H, d *J* = 8.50 Hz, Ar-H), 8.38 (1H, d *J* = 9.07 Hz, pyridine-H), 8.60 (1H, s, N=CH), 11.70 (1H, s, NH). ^13^C-NMR (75 MHz, DMSO-*d*_6_, ppm): 14.76 (CH_3_), 63.51 (CH_2_), 103.33 (Ar-CH), 122.1 (Ar-C), 129.05 (2CH, Ar-), 129.52 (2CH, Ar-), 133.94(Ar-C), 134.89, 138.64 (Ar-CH), 147.09 (N=CH), 149.47 (C-NH), 163.80 (C-F), 166.34 (C-OC_2_H_5_). IR (cm^−1^): ν_max_ 3365, 3300 (N-H stretching), 1640–1410 (C=N and C=C stretching), 1220–1050 (C-O). MS [M + 1]^+^: *m*/*z* 305.05.

*6-Ethoxy-2-[2-(4-methylbenzylidene)hydrazinyl]-3-nitropyridine* (**2d**). Yield: 85%, Melting point: 171 °C, ^1^H-NMR (300 MHz, DMSO-*d*_6_) δ (ppm): 1.40 (3H, t, CH_3_), 2.36 (3H, s, Ar-CH_3_) 4.52 (2H, q, CH_2_), 6.36 (1H, d *J* = 9.05 Hz, pyridine-H), 7.30 (2H, d J = 8.00 Hz, Ar-H), 7.65 (2H, d *J* = 8.04 Hz, Ar-H), 8.38 (1H, d *J* = 9.05 Hz, pyridine-H), 8.53 (1H, s, N=CH), 11.62 (1H, s, NH). ^13^C-NMR (75 MHz, DMSO-*d*_6_, ppm): 14.77 (CH_3_), 21.56 (CH_3_), 63.46 (CH_2_),103.06 (Ar-CH), 121.99 (Ar-C), 127.51 (2CH, Ar-), 129.98 (2CH, Ar-), 132.27 (Ar-C), 138.61 (Ar-CH), 140.35 (Ar-C), 148.60 (N=CH), 149.57 (C-NH), 166.38 (C-OC_2_H_5_). IR (cm^−1^): ν_max_ 3355, 3312 (N-H stretching), 1623–1388 (C=N and C=C stretching), 1225–1050 (C-O). MS [M + 1]^+^: *m*/*z* 301.

*6-Ethoxy-2-[2-(4-methoxybenzylidene)hydrazinyl]-3-nitropyridine* (**2e**). Yield: 80%, Melting point: 175 °C, ^1^H-NMR (300 MHz, DMSO-*d*_6_) δ (ppm): 1.40 (3H, t, CH_3_), 3.80 (3H, s, OCH_3_), 4.52 (2H, q, CH_2_), 6.34 (1H, d *J* = 9.03 Hz, pyridine-H), 7.05 (2H, d J = 8.72 Hz, Ar-H), 7.70 (2H, d *J* = 8.72 Hz, Ar-H), 8.35 (1H, d J = 9.07 Hz, pyridine-H), 8.52 (1H, s, N=CH), 11.60 (1H, s, NH). ^13^C-NMR (75 MHz, DMSO-*d*_6_, ppm): 14.77 (CH_3_), 55.79 (OCH_3_), 63.42 (CH_2_),102.93 (Ar-CH), 114.91 (2CH, Ar-), 121.64 (Ar-C), 127.52 (Ar-C), 129.14 (2CH, Ar-), 138.58 (Ar-CH), 148.50 (N=CH), 149.57 (C-NH), 161.34 (O-CH_3_), 166.41 (C-OC_2_H_5_). IR (cm^−1^): ν_max_ 3342, 3310 (N-H stretching), 1623-1408 (C=N and C=C stretching), 1200–1050 (C-O). MS [M + 1]^+^: *m*/*z* 318.12 (100%).

*6-Ethoxy-3-nitro-2-[2-(4-nitrobenzylidene)hydrazinyl]pyridine* (**2f**). Yield: 90%, Melting point: 281 °C, ^1^H-NMR (300 MHz, DMSO-*d*_6_) δ (ppm): 1.40 (3H, t, CH_3_), 4.52 (2H, q, CH_2_), 6.36 (1H, d *J* = 9.02 Hz, pyridine-H), 7.33 (2H, t *J* = 8.85 Hz, Ar-H), 7.70 (2H, t *J* = 8.85 Hz, Ar-H), 8.38 (1H, d *J* = 9.03 Hz, pyridine-H), 8.60 (1H, s, N=CH), 11.67 (1H, s, NH). ^13^C-NMR (75 MHz, DMSO-*d*_6_, ppm): 14.77 (CH_3_), 63.48 (CH_2_), 103.20 (Ar-CH), 116.41 (Ar-C-NO_2_), 116.58 (Ar-2CH), 129.57, 129.64 (Ar-2CH), 138.62 (Ar-CH), 139.02 (Ar-C), 147.33 (N=CH), 149.55 (Ar-C-NO_2_), 150.10 (Ar-C-NH), 168.50 (Ar-C-O). IR (cm^−1^): ν_max_ 3365, 3330 (N-H stretching), 1620–1410 (C=N and C=C stretching), 1200–1110 (C-O). MS [M + 1]^+^: *m*/*z* 332.

*6-Ethoxy-2-[2-(4-cyanobenzylidene)hydrazinyl]-3-nitropyridine* (**2g**). Yield: 83%, Melting point: 267 °C, ^1^H-NMR (300 MHz, DMSO-*d*_6_) δ (ppm): 1.40 (3H, t, CH_3_), 4.52 (2H, q, CH_2_), 6.42 (1H, d *J* = 9.17 Hz, pyridine-H), 7.90 (2H, d *J* = 8.66 Hz, Ar-H), 7.94 (2H, d *J* = 8.36 Hz, Ar-H), 8.40 (1H, d *J* = 9.04 Hz, pyridine-H), 8.65 (1H, s, N=CH), 11.80 (1H, s, NH). ^13^C-NMR (75 MHz, DMSO-*d*_6_, ppm): 14.76 (CH_3_), 63.57 (CH_2_), 103.72 (Ar-CH), 112.16 (Ar-C≡N), 119.20 (Ar-C-NO_2_), 122.70 (Ar-C), 127.89 (2CH, Ar), 133.33 (2CH, Ar), 138.66 (Ar-CH), 139.48 (Ar-C), 146.14 (N=CH), 149.28 (Ar-C-NH), 166.25 (C-OC_2_H_5_). IR (cm^−1^): ν_max_ 3365, 3330 (N-H stretching), 1620–1410 (C=N and C=C stretching), 1200–1110 (C-O). MS [M + 1]^+^: *m*/*z* 312.

*6-Ethoxy-2-[2-(4-dimethylaminobenzylidene)hydrazinyl]-3-nitropyridine* (**2h**). Yield: 89%, Melting point: 192 °C, ^1^H-NMR (300 MHz, DMSO-*d*_6_) δ (ppm): 1.40 (3H, t, CH_3_), 3.00 (6H, s, N(CH_3_)_2_, 4.52 (2H, q, CH_2_), 6.30 (1H, d *J* = 9.06 Hz, pyridine-H), 6.79 (2H, d *J* = 8.83 Hz, Ar-H), 7.59 (2H, d *J* = 8.79 Hz, Ar-H), 8.35 (1H, d *J* = 9.02 Hz, pyridine-H), 8.42 (1H, s, N=CH), 11.52 (1H, s, NH). ^13^C-NMR (75 MHz, DMSO-*d*_6_, ppm): 14.78 (CH_3_), 40.30 (2CH_3_, N-CH_3_), 63.36(CH_2_),102.64 (Ar-CH), 112.34 (2CH, Ar) 122.17 (Ar-C), 128.98(2CH, Ar), 132.3 (Ar-C),138.57 (Ar-CH), 149.49 (C=NH), 149.59 (C-NH), 152.05 (C-N(CH_3_)_2_), 166.47 (C-OC_2_H_5_). IR (cm^−1^): ν_max_ 3333, 3305 (N-H stretching), 1618–1410 (C=N and C=C stretching), 1217–1040 (C-O). MS [M + 1]^+^: *m*/*z* 330.

*6-Ethoxy-2-[2-(4-trifluoromethylbenzylidene)hydrazinyl]-3-nitropyridine* (**2i**). Yield: 85%, Melting point: 165 °C, ^1^H-NMR (300 MHz, DMSO-*d*_6_) δ (ppm): 1.21 and 1.40 (3H, d t, CH_3_), 4.31 and 4.52 (2H, d q, CH_2_), 6.38 (1H, d *J* = 9.20 Hz, pyridine-H), 7.81 (2H, d *J* = 8.80 Hz, Ar-H), 7.93 (2H, d *J* = 8.00 Hz, Ar-H), 8.36 (1H, d J = 9.20 Hz, pyridine-H), 8.64 (1H, s, N=CH), 10.91 and 11.71 (1H, d s, NH). ^13^C-NMR (75 MHz, DMSO-*d*_6_, ppm): 14.08 (CH_3_), 62.91 (CH_2_), 102.89 (CH), 121.85 (C), 125.57 (CH), 125.61 (C), 127.28 (CH), 129.25 (CH), 129.57 (CH), 137.93 (C), 138.15 (CH), 138.39 (C) 145.74 (C), 148.76 (N=CH), 165.65 (C). IR (cm^−1^): ν_max_ 3350, 3312 (N-H stretching), 1652–1430 (C=N and C=C stretching), 1220–1000 (C-O). MS [M + 1]^+^: *m*/*z* 355.10.

*4-[(2-(6-Ethoxy-3-nitropyridin-2-yl)hydrazono)methyl]phenol* (**2j**). Yield: 80%, Melting point: 158 °C, ^1^H-NMR (300 MHz, DMSO-*d*_6_) δ (ppm): 1.18 and 1.37 (3H, d t, CH_3_), 4.29 and 4.48 (2H, d q, CH_2_), 6.26 (1H, d *J* = 8.80 Hz, pyridine-H), 6.83 (2H, d *J* = 8.80 Hz, Ar-H), 7.56 (2H, d *J* = 8.40 Hz, Ar-H), 8.31 (1H, d *J* = 8.80 Hz, pyridine-H), 8.41 (1H, s, N=CH), 9.84 (1H, br, OH), 10.89 and 11.56 (1H, d s, NH). ^13^C-NMR (75 MHz, DMSO-*d*_6_, ppm): 14.11 (CH_3_), 62.78 (CH_2_), 102.14 (CH), 115.64 (C), 120.94 (2CH), 125.32 (CH), 128.72 (2CH), 137.88 (CH), 148.40 (N=CH), 148.97 (C), 159.35 (C-OH), 165.82 (C-O). IR (cm^−1^): ν_max_ 3340, 3320 (N-H stretching), 1657–1410 (C=N and C=C stretching), 1212–1005 (C-O). MS [M + 1]^+^: *m*/*z* 303.10 (100%).

*6-Ethoxy-2-[2-((1-methyl-1H-pyrrol-2-yl)methylene)hydrazinyl]-3-nitropyridine* (**2k**). Yield: 78%, Melting point: 163 °C, ^1^H-NMR (300 MHz, DMSO-*d*_6_) δ (ppm): 1.19 and 1.32 (3H, d t, CH_3_), 3.94 (3H, s, CH_3_), 4.29 and 4.44 (2H, d q, CH_2_), 6.09 (1H, t, pyrrole-H), 6.23 and 6.27 (1H, d d *J* = 9.20 Hz, pyridine-H), 6.45 (1H, m, pyridine-H), 6.94 (1H, br, pyrrole-H), 8.30 and 8.36 (1H, d d *J* = 8.80 Hz and 9.20 Hz, pyridine-H), 8.45 (1H, s, N=CH), 10.89 and 11.44 (1H, d s, NH). ^13^C-NMR (75 MHz, DMSO-*d*_6_, ppm): 14.13 (CH_3_), 36.14 (N-CH_3_) 62.52 (CH_2_), 102.06 (CH), 108.19 (CH), 115.6 (C), 120.70 (CH), 121.14 (CH), 128.32 (CH), 137.87 (C), 141.40 (N=CH), 148.84, (C), 165.76 (C-O). IR (cm^−1^): ν_max_ 3350, 3310 (N-H stretching), 1650–1430 (C=N and C=C stretching), 1225–1010 (C-O). MS [M + 1]^+^: *m*/*z* 290.12 (100%).

*6-Ethoxy-2-[2-((furan-2-yl)methylene)hydrazinyl]-3-nitropyridine* (**2l**). Yield: 77%, Melting point: 155 °C, ^1^H-NMR (300 MHz, DMSO-*d*_6_) δ (ppm): 1.17 and 1.33 (3H, d t, CH_3_), 4.29 and 4.45 (2H, d q, CH_2_), 6.28 and 6.37 (1H, d d *J* = 9.20 Hz, pyridine-H), 6.77 (1H, br, furan-H), 7.08 (1H, d *J* = 3.6 Hz, furan-H), 7.59 (1H, s, furan-H), 8.07 (1H, s, N=CH), 8.38 (1H, d *J* = 8.80 Hz, pyridine-H), 12.86 (1H, s, NH). ^13^C-NMR (75 MHz, DMSO-*d*_6_, ppm): 14.07 (CH_3_), 62.98 (CH_2_), 103.27 (CH), 112.39 (CH), 116.18 (C,), 121.75 (CH), 130.85 (CH), 137.99 (N=CH), 145.56 (CH), 147.63, (C), 149.32 (C), 166.06 (C). IR (cm^−1^): ν_max_ 3357, 3310 (N-H stretching), 1650–1435 (C=N and C=C stretching), 1225–1010 (C-O). MS [M + 1]^+^: *m*/*z* 277.09 (100%).

*6-Ethoxy-3-nitro-2-[2-((thiophen-2-yl)methylene)hydrazinyl]pyridine* (**2m**). Yield: 77%, Melting point: 152 °C, ^1^H-NMR (300 MHz, DMSO-*d*_6_) δ (ppm): 1.17 and 1.37 (3H, d t, CH_3_), 4.28 and 4.48 (2H, d q, CH_2_), 6.29 and 6.31 (1H, dd *J* = 8.8 Hz, *J* = 9.2 Hz pyridine-H), 7.12 (1H, t, thiophene-H), 7.39 (1H, d *J* = 3.2 Hz, thiophene-H), 7.62 (1H, d *J* = 5.2 Hz, thiophene-H) 8.33 and 8.38 (1H, dd *J* = 8.8 Hz, J = 9.2 Hz, pyridine-H), 8.75 (1H, s, N=CH), 10.85 and 11.59 (1H, d s, NH). IR (cm^−1^): ν_max_ 3345, 3312 (N-H stretching), 1656–1435 (C=N and C=C stretching), 1222–1000 (C-O). MS [M + 1]^+^: *m*/*z* 293.06 (100%).

*6-Ethoxy-3-nitro-2[(2-(pyridine-2-yl)methylene)hydrazinyl]pyridine* (**2n**). Yield: 85%, Melting point: 154 °C, ^1^H-NMR (300 MHz, DMSO-*d*_6_) δ (ppm): 1.17 and 1.39 (3H, d t, CH_3_), 4.28 and 4.51 (2H, d q, CH_2_), 6.28 and 6.47 (1H, dd *J* = 8.80 Hz, *J* =9.2 Hz pyridine-H), 8.08 (2H, d *J* = 6.8 Hz, pyridine-H), 8.37 and 8.39 (1H, dd *J* = 8.40 Hz, pyridine-H), 8.65 (1H, s, N=CH), 8.85 (1H, d *J* = 6.8 Hz, pyridine-H ), 10.68 and 12.02 (1H, d s, NH). IR (cm^−1^): ν_max_ 3345, 3305 (N-H stretching), 1650–1407 (C=N and C=C stretching), 1210–1020 (C-O). MS [M + 1]^+^: *m*/*z* 288.10 (100%).

### 3.3. Biological Activity

#### 3.3.1. MAO Activity Assay

The enzyme activity assay was performed in accordance with the procedure reported by Matsumoto et al. [15] with slight modifications. All materials used in the enzymatic assays were purchased from Sigma-Aldrich Chemicals. All pipetting protocols in the assay were performed by the Biotek Precision robotic system (BioTek Instruments, Inc., Winooski, VT, USA). To prevent the effect of of light MAO enzymes, the assay was performed in 96-well black plates. Phosphate buffer (0.1 M, pH = 7.4) was used for the preparation of 5 mg/mL stock solutions of the human recombinant MAO-A and MAO-B enzymes. The enzyme stock solutions were further diluted with an assay buffer to obtain final concentrations of 0.006 mg/mL MAO-A and 0.015 mg/mL MAO-B. Kynuramine was dissolved in sterilized distilled water to obtain a 25 mM stock solution and then diluted with the assay buffer to obtain final concentrations of 40 μM for the MAO-A enzyme and 20 μM for the MAO-B enzyme. The synthesized compounds and reference drugs (moclobemide for the MAO-A enzyme and selegiline for the MAO-B enzyme) were diluted to 10^−3^ M and 10^−4^ M concentrations in 2% DMSO and added at 100 μL/well. The addition of the diluted compounds was followed by the addition of either the MAO-A or MAO-B enzymes (50 μL/well). After incubation for 10 min at 37 °C, kynuramine (50 μL/well) was added to initiate the enzyme-substrate reaction. The plate was incubated for 20 min at 37 °C and the reaction was subsequently terminated by the addition of 2 N NaOH (75 μL/well). Fluorescence was read from top by using BioTek-Synergy H1 multimode microplate reader at the excitation/emission wavelengths of 310 and 380 nm. The same procedure was followed for the dilutions (10^−5^–10^−9^ M, 100 μL/well) of the inhibitors and selected compounds that indicated ≥50% inhibition at the initial concentrations (10^−3^ and 10^−4^ M, 100 μL/well) (Table 1). 

The IC_50_ values (Table 2) were calculated from the plots of enzyme activity against concentrations, and the regression analyses were computed in GraphPad Prism Version 6 (GraphPad Software, Inc., La Jolla, CA, USA).

#### 3.3.2. Enzyme Kinetic Studies

The same materials were used for the MAO-inhibition assay. Compound **2j** was prepared at the IC_50_ concentration calculated in the enzyme assay and then added to the wells (100 μL/well). The stock solution (25 mM) of kynuramine in 0.1 M phosphate buffer was diluted to final concentrations of 40, 20, 10, 5, 2.5 and 1.25 μM and then added to the wells (50 μL/well) that contained the test compound. The MAO-A enzyme was added to the plate (50 μL/well), incubated for 20 min at 37 °C, and the plate was read at excitation/emission wavelengths of 310 and 340 nm [20]. The control measurement without the inhibitor was determined simultaneously. The results were analyzed by using Lineweaver-Burk plots in Microsoft Office Excel 2013 (Microsoft Inc., Redmond, WA, USA).

### 3.4. Cytotoxicity Test

For the cytotoxicity test, NIH/3T3 mouse embryonic fibroblast cells (ATCC^®^ CRL-1658™, London, UK) were first incubated in Dulbecco’s modified Eagle’s medium (DMEM), plated in 96-well culture plates at 10,000 cells/well, and then treated with the compounds at concentrations between 1 mM and 0.000316 mM (1, 0.316, 0.1, 0.0316, 0.01, 0.00316, 0.001, 0.000316 mM). The MTT assay was performed as previously described [21,22,23,24,25,26]. The formula below was used to calculate the inhibition percentage for each concentration, and the percentages inhibition of cell proliferation was used to calculate the IC_50_ value:

% inhibition = 100 − (mean sample × 100/mean solvent)(1)

### 3.5. Molecular Docking Studies

A structure based in silico procedure was applied to determine the binding modes of the compound **2j** *h*MAO-A enzyme active site. The crystal structures of *h*MAO-B (PDB ID: 2Z5X) [17], which was crystallized with the reversible inhibitor safinamide, was retrieved from the Protein Data Bank server (www.pdb.org (access date: 14 November 2017)).

The structures of ligands were built using the Schrödinger Maestro [27] interface and were then submitted to the Protein Preparation Wizard protocol of the Schrödinger Suite 2016 Update 2 [28]. The ligands were prepared by the LigPrep 3.8 [29] to correctly assign the protonation states at pH 7.4 ± 1.0 and the atom types. The bond orders were assigned, and the hydrogen atoms were added to the structures. The grid generation was formed by using Glide 7.1 [30]. The grid box with dimensions of 20 Å × 20 Å × 20 Å, was centered in the vicinity of the flavin (FAD) N5 atom on the catalytic site of the protein to cover all binding sites and neighboring residues [31,32,33]. The flexible docking runs were performed in standard precision docking mode (SP).

## 4. Conclusions 

Interest in MAO inhibitors has increased in recent years owing their restorative influence on mental health [3], therefore, a novel series of *N*-pyridyl-hydrazone derivatives was synthesized, starting from 2-chloro-6-ethoxy-3-nitropyridine, and their structures were confirmed by FT-IR, ^1^H-NMR, ^13^C-NMR and ESI mass spectroscopy. These compounds show strong and specific inhibitory activity against MAO-A and MAO-B enzymes; compounds **2i**, **2j**, **2k**, **2l** and **2n** exhibited the highest activity with IC_50_ values of 13.06 μM, 6.25 μM, 6.12 μM, 10.64 μM and 9.52 μM, respectively, against MAO A, whereas compound **2j** exhibited the strongest MAO-B inhibitory activity, with a IC_50_ value of 9.30 μM. Furthermore, these active compounds did not indicate any cytotoxic effects at the dose required for MAO inhibition. This work is in progress and future developments are expected. We have presented all the results on the figureMAO-A and MAO-B inhibition studies, as they may have potential effects on the future remedial treatment of neurodegenerative diseases. In addition, these derivatives may provide possible therapeutic options for the treatment of the motor and non-motor symptoms of Parkinson’s disease, for which no treatment has been previously demonstrated. 

## Figures and Tables

**Table 1:** Inhibitory activity (%) ± S.D. of the compounds (**2a**–**2n**), moclobemide and selegiline against MAO-A and MAO-B enzymes.

Compound	MAO-A Inhibition %		MAO-B Inhibition %	
	10^−3^ M	10^−4^ M	10^−3^ M	10^−4^ M
**2a**	25.63 ± 0.88	17.85 ± 0.68	0.76 ± 0.03	0.58 ± 0.01
**2b**	48.97 ± 1.22	0.46 ± 0.01	0.43 ± 0.02	0.11 ± 0.004
**2c**	51.95 ± 1.02	7.09 ± 0.18	60.21 ± 1.18	0.69 ± 0.03
**2d**	20.14 ± 0.41	11.41 ± 0.28	0.52 ± 0.02	0.18 ± 0.006
**2e**	55.61 ± 1.05	13.96 ± 0.41	33.09 ± 1.14	0.22 ± 0.008
**2f**	64.98 ± 1.23	16.02 ± 0.56	7.39 ± 0.31	0.55 ± 0.02
**2g**	25.86 ± 0.87	14.42 ± 0.54	0.67 ± 0.03	0.27 ± 0.01
**2h**	80.78 ± 1.31	16.93 ± 0.49	86.28 ± 1.25	3.52 ± 0.15
**2i**	55.72 ± 1.26	51.70 ± 1.18	38.28 ± 1.19	29.12 ± 0.99
**2j**	75.67 ± 1.85	69.76 ± 1.43	63.77 ± 1.28	52.69 ± 1.29
**2k**	76.56 ± 1.38	66.08 ± 1.47	40.91 ± 1.02	39.73 ± 1.15
**2l**	67.80 ± 1.44	64.43 ± 1.30	51.00 ± 1.08	43.05 ± 1.06
**2m**	41.42 ± 1.13	36.05 ± 1.15	34.42 ± 0.88	21.17 ± 0.80
**2n**	72.07 ± 1.56	56.90 ± 1.50	42.00 ± 1.21	25.29 ±1 .08
**Moclobemide**	94.12 ± 2.76	82.14 ± 2.69	-	-
**Selegiline**	-	-	98.94 ± 2.06	94.73 ± 1.97

**Table 2:** IC_50_ values of compounds **2i**, **2j**, **2k**, **2l**, **2n**, moclobemide and selegiline against MAO-A and MAO-B.

Comp.	MAO-A Inhibition %							MAO-A IC_50_ (µM)
	10^−3^ M	10^−4^ M	10^−5^ M	10^−6^ M	10^−7^ M	10^−8^ M	10^−9^ M	
**2i**	55.72 ± 1.26	51.70 ± 1.18	31.19 ± 1.20	21.08 ± 0.84	15.45 ± 0.62	12.66 ± 0.43	11.57 ± 0.38	13.06
**2j**	75.67 ± 1.85	69.76 ± 1.43	56.12 ± 1.27	43.18 ± 1.06	38.77 ± 1.07	32.68 ± 0.82	26.74 ± 0.96	6.25
**2k**	76.56 ± 1.38	66.08 ± 1.47	54.72 ± 1.20	36.58 ± 1.08	33.23 ± 0.92	25.20 ± 0.88	22.99 ± 0.89	6.12
**2l**	67.80 ± 1.44	64.43 ± 1.30	42.97 ± 1.02	31.01 ± 0.98	27.17 ± 0.89	19.75 ± 0.41	17.88 ± 0.17	10.64
**2n**	72.07 ± 1.56	56.90 ± 1.50	42.13 ± 1.22	35.11 ± 1.18	24.96 ± 0.83	18.02 ± 0.77	10.40 ± 0.29	9.52
**Moclobemide**	94.12 ± 2.76	82.14 ± 2.69	60.45 ± 2.55	36.15 ± 1.98	22.13 ± 1.33	18.16 ± 0.81	14.12 ± 0.72	6.06
**Comp.**	**MAO-B Inhibition %**							**MAO-B IC_50_ (µM)**
	**10^−3^ M**	**10^−4^ M**	**10^−5^ M**	**10^−6^ M**	**10^−7^ M**	**10^−8^ M**	**10^−9^ M**	
**2j**	63.77 ± 1.28	52.69 ± 1.29	40.22 ± 1.12	31.60 ± 1.08	25.00 ± 0.95	21.93 ± 0.82	12.48 ± 0.51	9.30
**Selegiline**	98.94 ± 2.06	94.73 ± 1.97	86.96 ± 1.82	79.22 ± 1.71	65.36 ± 1.12	43.27 ± 1.02	14.70 ± 0.25	0.038

## Supplementary Materials

The following are available online.
